# Supplementary material for: Dependence of contextual modulation in macaque V1 on interlaminar signal flow
Source: eLife. 2026 Jan 6;13:RP103255. doi: 10.7554/eLife.103255 (PMC12774416; doi:10.7554/eLife.103255)
Supplement: Supplementary file 1. [file elife-103255-supp1.docx]

**Supplementary File 1. Number of neurons selective to B_own_ (LC) across recordings.**

| **Layer** | Total | **p_Side_**  **<0.05** | | **p_Side_**  **≥0.05** | | **Layer** | Total | **p_Side_**  **<0.05** | | **p_Side_**  **≥0.05** | |
| --- | --- | --- | --- | --- | --- | --- | --- | --- | --- | --- | --- |
|  |  | p_LC_  ≥0.05 | p_LC_ <0.05 | p_LC_  <0.05 | p_LC_ ≥0.05 |  |  | p_LC_  ≥0.05 | p_LC_ <0.05 | p_LC_  <0.05 | p_LC_ ≥0.05 |
|  | ***pen*1** | | | | |  | ***pen2*** | | | | |
| 2/3 | 29 | 6 | 5 | 6 | 12 | 2/3 | 55 | 1 | 14 | 18 | 22 |
| 4A/B | 19 | 0 | 5 | 8 | 6 | 4A/B | 34 | 0 | 7 | 10 | 17 |
| 4C | 38 | 3 | 7 | 16 | 12 | 4C | 54 | 4 | 15 | 22 | 13 |
| 5/6 | 40 | 2 | 7 | 18 | 13 | 5/6 | 27 | 5 | 2 | 4 | 16 |
|  | ***pen3*** | | | | |  | ***pen4*** | | | | |
| 2/3 | 15 | 1 | 1 | 4 | 9 | 2/3 | 0 | 0 | 0 | 0 | 0 |
| 4A/B | 27 | 0 | 4 | 9 | 14 | 4A/B | 34 | 3 | 0 | 2 | 29 |
| 4C | 60 | 1 | 12 | 24 | 23 | 4C | 60 | 2 | 4 | 5 | 49 |
| 5/6 | 40 | 2 | 7 | 2 | 29 | 5/6 | 37 | 3 | 0 | 2 | 32 |
|  | ***pen all*** | | | | |  |  |  |  |  |  |
| 2/3 | 99 | 8 | 20 | 28 | 43 |  |  |  |  |  |  |
| 4A/B | 114 | 3 | 16 | 29 | 66 |  |  |  |  |  |  |
| 4C | 212 | 10 | 38 | 67 | 97 |  |  |  |  |  |  |
| 5/6 | 144 | 12 | 16 | 26 | 90 |  |  |  |  |  |  |
