# Supplementary material for: Dependence of contextual modulation in macaque V1 on interlaminar signal flow
Source: eLife. 2026 Jan 6;13:RP103255. doi: 10.7554/eLife.103255 (PMC12774416; doi:10.7554/eLife.103255)
Supplement: Supplementary file 2. [file elife-103255-supp2.docx]

**Supplementary File 2. Comparison of CCG asymmetries during CRF and nCRF for each recording.**

| **4C-2/3** | *CRF* | *nCRF* | **Δ** | ***p*** | **4A/B-2/3** | *CRF* | *nCRF* | **Δ** | ***p*** |
| --- | --- | --- | --- | --- | --- | --- | --- | --- | --- |
| *pen*1 | 0.036 | -0.004 | -0.040 | 2.8*10^-7^ | *pen*1 | 0.046 | -0.005 | -0.050 | 7.5*10^-12^ |
| *pen2* | 0.037 | 0.013 | -0.024 | 3.7*10^-9^ | *pen2* | 0.030 | 0.022 | -0.008 | 0.066 |
| *pen3* | 0.017 | 0.033 | 0.016 | 0.29 | *pen3* | 0.049 | 0.052 | 0.003 | 0.99 |
| **4C-5/6** | *CRF* | *nCRF* | **Δ** | ***p*** | **4A/B-5/6** | *CRF* | *nCRF* | **Δ** | ***p*** |
| *pen*1 | -0.010 | -0.044 | -0.033 | 0.0015 | *pen*1 | 0.012 | -0.028 | -0.040 | 2.1*10^-6^ |
| *pen2* | -0.009 | -0.027 | -0.018 | 1.5*10^-5^ | *pen2* | -0.011 | -0.018 | -0.007 | 0.059 |
| *pen3* | -0.036 | -0.062 | -0.026 | 5.8*10^-7^ | *pen3* | -0.025 | -0.087 | -0.063 | 9.1*10^-15^ |
| *pen4* | -0.041 | -0.093 | -0.051 | 3.1*10^-8^ | *pen4* | -0.030 | -0.089 | -0.060 | 9.1*10^-12^ |
