# Supplementary material for: Dependence of contextual modulation in macaque V1 on interlaminar signal flow
Source: eLife. 2026 Jan 6;13:RP103255. doi: 10.7554/eLife.103255 (PMC12774416; doi:10.7554/eLife.103255)
Supplement: Supplementary file 3. [file elife-103255-supp3.docx]

**Supplementary File 3. GLMs for dependence of border ownership and local contrast on CCG asymmetry.**

$$B_{own}\left( pair \right)\sim1+CCG asymmetry+ laminar combination+pen$$

$$LC\left( pair \right)\sim1+CCG asymmetry+ laminar combination+pen$$

| GLM | Border ownership (B_own_) | | | | Local contrast (LC) | | | |
| --- | --- | --- | --- | --- | --- | --- | --- | --- |
|  | Estimate | SE | tStat | p | Estimate | SE | tStat | p |
| Intercept | 0.166 | 0.008 | 21.379 | 0 | 0.362 | 0.010 | 35.352 | 0 |
| **CCG asymmetry** | **-0.158** | 0.032 | -4.880 | **1.1*10^-6^** | **-0.044** | 0.043 | -1.037 | **0.300** |
| 4C-5/6  *_rel.4C-2/3_* | 0.014 | 0.007 | 1.948 | 0.05 | -0.117 | 0.009 | -12.606 | 3.5*10^-35^ |
| 4A/B-2/3 *_rel.4C-2/3_* | 2.5*10^-5^ | 0.006 | 0.004 | 0.99 | -0.016 | 0.008 | -1.877 | 0.061 |
| 4A/B-5/6 *_rel.4C-2/3_* | 0.009 | 0.007 | 1.351 | 0.18 | -0.136 | 0.009 | -15.093 | 0 |
| *pen2_rel.pen1_* | -0.009 | 0.007 | -1.219 | 0.22 | 0.010 | 0.010 | 0.980 | 0.327 |
| *pen3 _rel.pen1_* | 0.038 | 0.009 | 4.435 | 9.7*10^-6^ | 0.035 | 0.011 | 3.082 | 0.002 |
| *pen4 _rel.pen1_* | 0.049 | 0.010 | 5.147 | 2.9*10^-7^ | -0.088 | 0.013 | -6.910 | 6.4*10^-12^ |

Number of observations: 2118; Error degrees of freedom: 2110

GLM(B_own_): R^2^=0.11; F-statistic vs. constant model: 37.2, p-value=0.

GLM(LC): R^2^=0.27; F-statistic vs. constant model: 109, p-value=0.
